# Differentiated regulation of immune-response related genes between LUAD and LUSC subtypes of lung cancers

**DOI:** 10.18632/oncotarget.13346

**Published:** 2016-11-15

**Authors:** Mengzhu Chen, Xiuying Liu, Jie Du, Xiu-Jie Wang, Lixin Xia

**Affiliations:** ^1^ State Key Laboratory of Respiratory Disease for Allergy at Shenzhen University, Department of Biochemistry and Molecular Biology, Health Science Center of Shenzhen University, School of Medicine, Shenzhen University, Guangdong 518060, China; ^2^ Key Laboratory of Genetic Network Biology, Collaborative Innovation Center of Genetics and Development, Institute of Genetics and Developmental Biology, Chinese Academy of Sciences, Beijing 100101, China; ^3^ Beijing Anzhen Hospital, Capital Medical University, Beijing Collaborative Innovative Center for Cardiovascular Disorders, The Key Laboratory of Remodeling-Related Cardiovascular Diseases, Ministry of Education, Beijing Institute of Heart Lung and Blood Vessel Diseases, Beijing 100029, China

**Keywords:** LUAD, LUSC, immune-response related genes, expression features, cancer progression rate

## Abstract

Lung squamous cell carcinoma (LUSC) and lung adenocarcinoma (LUAD) are the two major subtypes of lung cancer, with LUSC exhibits faster progression rate than LUAD. To investigate the roles of immune-response related genes (IRGs) in lung cancer progression, we used LUAD and LUSC samples at different cancer progression stages, and identified that the expression profiles of IRGs could serve as a better classification marker for cancerous tissues of both LUAD and LUSC. We found that the expression changes of IRGs were different between LUAD and LUSC. Cell cycle promoting genes, including KIFs and proteasomes, showed faster up-regulation in LUSC, whereas immune response promoting genes, including MHC molecules and chemokines, were more rapidly repressed in LUSC. Comparative pathway analysis revealed that members of the Toll-like receptor and T cell receptor signaling pathways exhibited diverged expression changes between LUAD and LUSC, especially at the early cancer stages. Our results revealed the difference of LUAD and LUSC from the immune response point of view, and provided new clues for the differential treatment of LUAD and LUSC.

## INTRODUCTION

Lung cancer is a common and severe disease which ranks the top among cancers worldwide in terms of mortality for both men and women [1, 2]. Most of diagnosed lung cancers are malignant epithelial tumors, which could be further classified into small cell lung carcinoma (SCLC) and non-small cell lung carcinoma (NSCLC). NSCLC counts for about 85-90% of lung cancers, among which the most common subtypes are lung adenocarcinoma (LUAD) and lung squamous cell carcinoma (LUSC) [3]. According to the tumor node metastasis (TNM) taxonomy, both LUAD and LUSC can be classified into four stages, namely stages I, II, III and IV [4]. Stage I refers to the early, non-metastatic stage. Stages II and III usually indicate the intermediate, regional lymphatic metastatic stages, of which stage III has a higher lymphatic metastasis degree than stage II. And stage IV usually represents the late stage with distal metastasis. Each stage can be further divided into A and B sub-stages, of which sub-stage B has higher cancer degree than sub-stage A.

LUAD is at present the most common lung cancer subtype among non-smokers and women, although it has been shown that smoking may increase the risk of LUAD [5, 6]. By contrast, LUSC is closely associated with smoking, and is more common in men than in women [7]. Generally LUAD grows more slowly with smaller masses than LUSC of the same stage, but LUAD tends to initiate metastasis at the early stages [8]. Pan-cancer studies have shown that the molecular mechanisms of carcinogenesis could be highly heterogeneous between LUAD and LUSC, even in LUAD itself [3, 9, 10]. Consequently, therapies for LUAD are often ineffective for LUSC [11].

A number of genes have been reported to be associated with lung cancer, including *EGFR*, *TP53*, *AKT1*, *DDR2*, *FGFR1*, *KRAS*, *PTEN*, and others [12–18]. Previous studies have shown that the patients' immune system plays a key role in controlling the development of lung cancer [19]. Cancer cells with abnormal mutation could provoke the body's immune responses therefore to be identified and eliminated. It has been shown that animals with deficiency in key immune response related genes are more prone to develop cancers [20, 21]. On the other hand, cancer cells have also acquired the ability to alter host immune response to facilitate cancer progression [22–25]. Although methods have been developed to diagnose or predict the clinical treatment outcomes of lung cancers basing on the expression profiles of certain immune-response related genes (IRGs) [26–28], thorough comparison of the expression changes of IRGs and their participated pathways/networks during the progression process of LUAD and LUSC is still lacking.

Here, we systematically studied the expression changes of IRGs during the progression process of LUAD and LUSC. Through comparative analysis, we have revealed the expression difference of IRGs between LUAD and LUSC, and identified genes and pathways that may contribute to the faster progression rate of LUSC in comparison to LUAD.

## RESULTS

### Immune-response related genes (IRGs) showed systematic expression changes in the LUAD and LUSC samples

**Figure 1:**
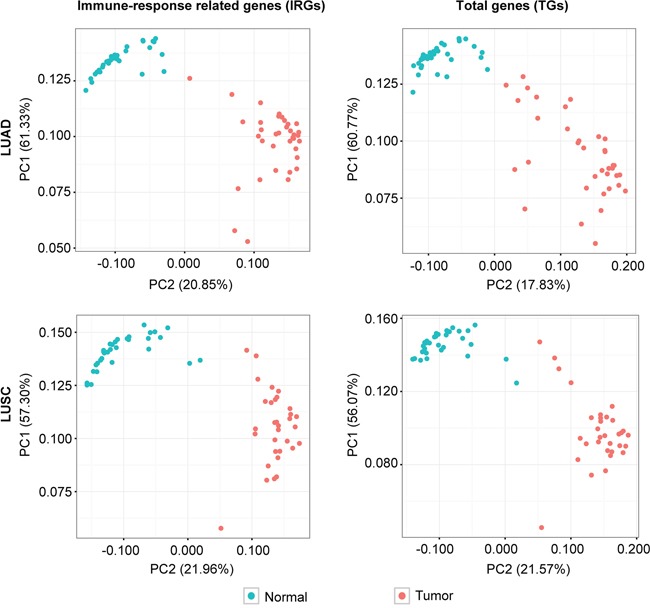
Principle component analysis (PCA) of the expression levels of immune-response related genes (IRGs) and total genes (TGs) among the tumor (red points) and normal (blue points) samples from the LUAD and LUSC patients, respectively Percentages in parenthesis are the proportion of variability presented by each PCA.

To investigate the expression changes of immune-response related genes (IRGs) from the LUAD and LUSC subtype cancer samples, we collected the RNA-Seq data of LUAD and LUSC patients from The Cancer Genome Atlas (TCGA) database. We considered patients with available RNA-seq data from both the tumor and matched normal samples as valid subjects, thus, data from a total of 36 LUAD patients and 32 LUSC patients at different cancer stages were included in this study (Supplementary Table S1 and Supplementary Table S2). We extracted the expression information of IRGs from each sample according to the list of IRGs curated by the Immunology Database and Analysis Portal (IMMPORT) website, which includes genes directly or indirectly involved in immune responses. A total of 6001 IRGs were curated by the IMMPORT dataset, among which, around 4100 were detected in each stage of the LUAD and LUSC samples, respectively (Table 1). Principle component analysis (PCA) showed that the expression profiles of total expressed IRGs could better classify the tumor and normal samples than the profiles of the total expressed genes for both the LUAD and LUSC patients (Figure 1), indicating that the IRGs had undergone systematic and consistent expression changes among the tumor tissues of both LUAD and LUSC.

**Table 1:** No. of expressed immune-response related genes (IRGs) and differentially expressed IRGs of the LUAD and LUSC samples at different cancer stages

Dataset	Category of IRGs		Total	Cancer stage				
				IA	IB	II	III	IV
LUAD	Expressed		4238	4158	4172	4185	4166	4082
	Differentially expressed	Up	1336	561	770	896	738	160
		Down	1048	551	686	570	746	119
LUSC	Expressed		4252	4177	4201	4199	4167	4015
	Differentially expressed	Up	1622	897	1029	1094	850	574
		Down	1405	678	1016	950	552	519

Up, up-regulated genes in tumor samples in comparison to normal samples;

Down, down-regulated genes in tumor samples in comparison to normal samples.

Using a cutoff threshold of fold change > 2 and FDR < 0.1 (with Benjamini–Hochberg multiple testing correction), differentially expressed immune-response related genes (DEIRGs) at each stage of LUAD or LUSC as compared to the corresponding normal tissues were identified by the edgeR software and used in the following analysis (Table 1). Comparative analysis revealed that more than 59% of the DEIRGs with unidirectional expression changes (see materials and methods) presented in both LUAD and LUSC (Supplementary Figure S1). GO analysis of the common DEIRGs with unidirectional expression changes revealed that genes involved in multiple immune related processes were down-regulated in both LUAD and LUSC, indicating that the development of both LUAD and LUSC is accompanied by the repression of patient immune responses (Supplementary Figure S2). To the contrary, cell cycle and cell division related genes were up-regulated, which was in concert with the fast proliferation feature of cancer cells (Supplementary Figure S2). In addition, more immune related GO terms were enriched among LUSC specifically repressed genes as compared to those of LUAD, which may partially contribute to the faster progression process of LUSC (Supplementary Figure S3).

### Expression pattern changes of DEIRGs during cancer progression

**Figure 2:**
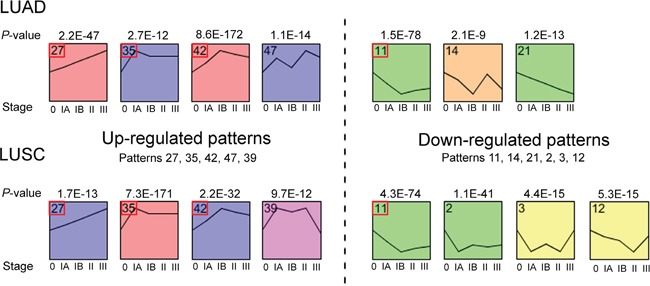
Schematic diagrams of the significantly enriched expression patterns of the differentially expressed immune-response related genes (DEIRGs) during LUAD or LUSC progression Patterns present in both LUAD and LUSC samples are marked with red boxes.

**Figure 3:**
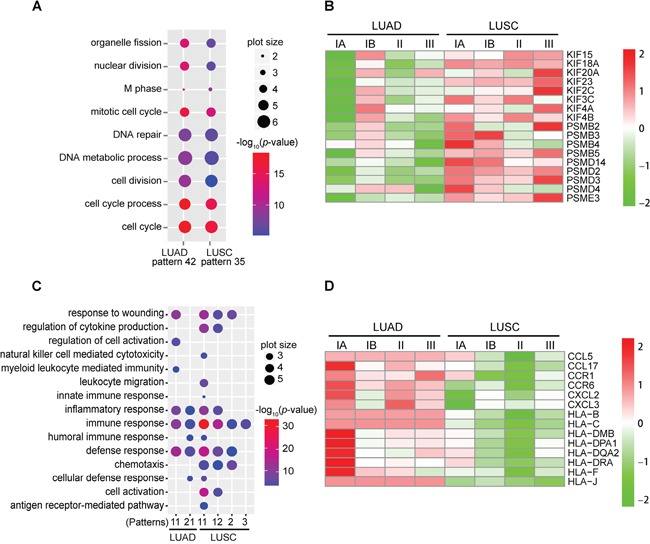
Functional analysis of DEIRGs with significantly enriched expression patterns **A.** Commonly enriched GO terms of DEIRGs with pattern 42 of LUAD and pattern 35 of LUSC. Shown are significantly enriched GO terms (FDR < 0.001, Fisher's exact test) of the biological process category. Dot size represents the frequency of the GO term in the Gene Ontology annotation (GOA) database. Dot color represents the log_10_-transformed enrichment *p*-value of each GO term. **B.** Expression changes of the KIFs and proteasome genes during LUAD and LUSC progression. The scaled relative abundances of log_2_-transformed RPKM ratio (tumor/normal) values are shown in the heatmap. **C.** Commonly enriched GO terms of DEIRGs with down-regulated expression patterns among LUAD and LUSC samples. Shown are the significantly enriched GO terms (FDR < 0.001, Fisher's exact test) of the biological process category. Pattern 14 of LUAD is not included as no enriched GO terms were identified among genes of this pattern. **D.** Expression changes of the MHC and chemokine genes during LUAD and LUSC progression. The scaled relative abundances of log_2_-transformed RPKM ratio (tumor/normal) values are shown in the heatmap.

To investigate the expression changes of DEIRGs during the progression of LUAD and LUSC, we classified the expression patterns of DEIRGs using the STEM (short time-series expression miner) software [29]. A total of 7 and 8 significantly enriched expression patterns were identified among DEIRGs of LUAD and LUSC, respectively (*p*-value < 0.05, non-parametric clustering algorithm of STEM with Bonferroni correction) (Figure 2 and Supplementary Table S3). Patterns 11, 27, 35 and 42 were identified among DEIRGs of both LUAD and LUSC, whereas patterns 14, 21 and 47 only presented among DEIRGs of LUAD, and patterns 2, 3, 12 and 39 were only detected among DEIRGs of LUSC (Figure 2). By comparing the gene expression level at the most significantly altered cancer stage to that of the normal samples, we roughly divided these patterns into up-regulated and down-regulated expression groups in both LUAD and LUSC (Figure 2). Unexpectedly, only few common genes were identified among the groups with identical patterns in LUAD and LUSC (Supplementary Table S3). Comparative GO analysis revealed that the enriched biological processes among DEIRGs with pattern 42 of LUAD were similar to those among DEIRGs with pattern 35 of LUSC (Supplementary Figure S4), and most of these processes were related to cell proliferation, cell cycle and DNA repair processes (Figure 3A). The expression of genes with pattern 35 reached the peak level at stage IA, whereas the expression of genes with pattern 42 reached the peak level at stage IB. Such earlier activation of cell proliferation and cell cycle related genes in LUSC as compared to LUAD might be one of the causal factors for the faster progression of LUSC. Correspondingly, at stage IA, the expression levels of cell proliferation and cell cycle related genes with pattern 42 of LUAD were generally lower than those with pattern 35 of LUSC (Supplementary Table S4).

To be specific, we found that kinesin superfamily genes (*KIF*s) and proteasome complex subunit genes (*PSMB*s, *PSMD*s and *PSME3*) were among genes with pattern 42 of LUAD and pattern 35 of LUSC (Figure 3B and Supplementary Table S4). It has been shown that KIFs play important roles in tumor development and progression due to their crucial functions in regulating cell division as well as intracellular vesicle and organelle transportion [30, 31]. Malfunction of the proteasome complex could also contribute to the pathogenesis of cancer [32–34].

On the other hand, immune response processes related GO terms were significantly enriched among DEIRGs with down-regulated expression patterns, including patterns 11 and 21 of LUAD, and patterns 2, 3, 11, and 12 of LUSC (Figure 3C, Supplementary Figure S5, and Supplementary Table S5). We noticed that MHC molecules and chemotactic cytokines were overrepresented among the DEIRGs with down-regulated patterns. MHC molecules are crucial for mediating antigen processing and presentation during immune responses [22, 35, 36]. Chemokine can guide the migration of cells, such as directing lymphocytes to the lymph nodes to provoke immune response during immune surveillance [37]. The expression levels of the above mentioned two types of genes at the early stage of LUSC were generally lower than those of LUAD (Figure 3D and Supplementary Table S5), implying their contribution to the faster tumor growth rate of LUSC by escaping the immune surveillance.

### Comparative pathway analysis of DEIRGs during LUAD and LUSC progression

**Figure 4:**
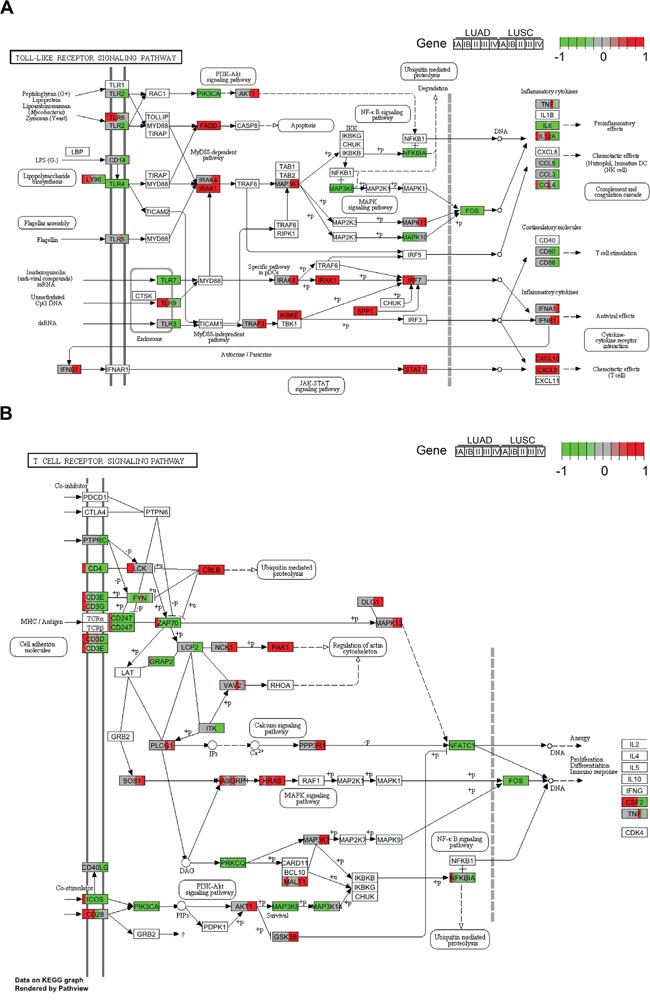
Expression changes of the Toll-like receptor and T cell receptor signaling pathway genes during LUAD and LUSC progression Each gene box is equally divided into ten pieces, sequentially representing the five stages (IA, IB, II, III, and IV) of LUAD and LUSC. Colors represent the scaled relative abundances of log_2_-transformed RPKM ratio (tumor/normal) values. **A.** Expression changes of DEIRGs in the Toll-like receptor signaling pathway. **B.** Expression changes of DEIRGs in the T cell receptor signaling pathway.

To thoroughly compare the signaling pathways in which DEIRGs participated during LUAD and LUSC progression, we carried out enriched signaling pathway analysis of all DEIRGs with unidirectional expression changes in LUAD and LUSC. Among the identified pathways, Toll-like receptor (TLR) and T cell receptor signaling pathways exhibited synergetic expression differences between LUAD and LUSC. For the TLR pathway, both the upstream *TLR*s (including *TLR2*, *TLR3*, *TLR4*, *TLR5*, *TLR7* and *TLR9*) and the downstream effector genes (including *CCL3*, *CCL4* and *CCL5* in the complement and coagulation cascade, and *CD80* and *CD86* with T cell stimulation functions) all exhibited reduced expression in LUSC as compared with LUAD (Figure 4A). Similarly, genes involved in T cell mediated immune response, such as *CD3D*, *CD3E*, *CD3G*, *CD247* in the CD3-TCR complex and downstream effector *ZAP70*, were more rapidly repressed in LUSC than in LUAD, especially at the early cancer stage (Figure 4B).

### DEIRGs with diverged and stage-specific expression patterns

**Figure 5:**
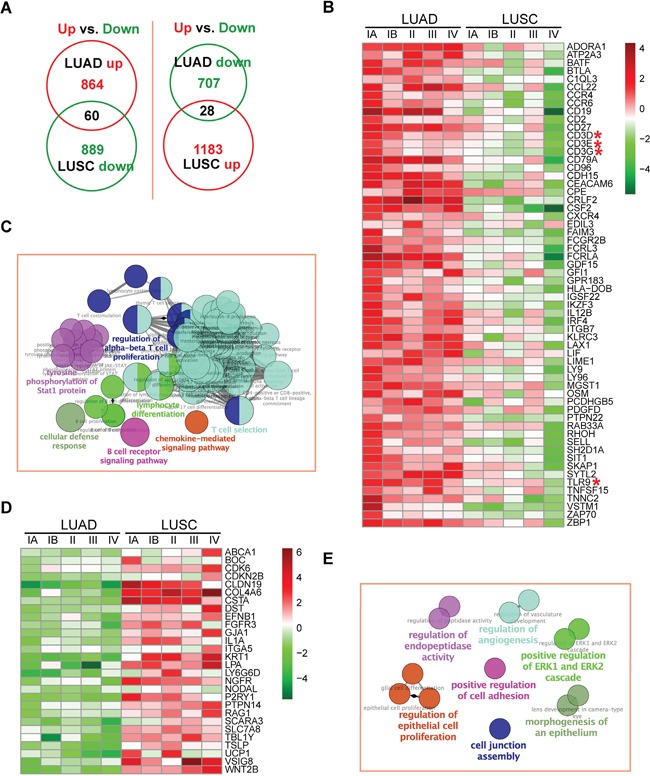
Expression profiles and GO analysis of DEIRGs with diverged changes between LUAD and LUSC **A.** Venn diagram analysis of DEIRGs with unidirectional expression changes in LUAD and LUSC. **B.** Expression profiles of DEIRGs up-regulated in LUAD and down-regulated in LUSC. Log_2_-transformed RPKM ratio (tumor/normal) values are shown in the heatmap. The CD3-TCR complex members and Toll-like receptor were highlighted with red asterisks. **C.** Enriched GO terms (*p*-value < 0.01, corrected with Bonferroni step down) of DEIRGs in panel B. The names of processes and their related GO terms are shown in the same colors. Circles are connected according to the hierarchical relationships of GO terms. The sizes of circles are negatively correlated with the enrichment *p*-values of GO terms. **D.** Expression profiles of DEIRGs down-regulated in LUAD and up-regulated in LUSC. Log_2_-transformed RPKM ratio (tumor/normal) values are shown in the heatmap. **E.** Enriched GO terms (*p*-value < 0.05, corrected with Bonferroni step down) of DEIRGs in panel D.

Next, we searched for DEIRGs exhibited diverged expression changes in LUAD and LUSC. By searching for DEIRGs with unidirectional up-regulation in one cancer subtype whereas with unidirectional repression in the other cancer subtype, we identified 60 DEIRGs being up-regulated in LUAD but repressed in LUSC as compared to their corresponding normal tissues (Figure 5A and 5B and Supplementary Table S6), as well as 28 genes being repressed in LUAD but up-regulated in LUSC (Figure 5A and 5D and Supplementary Table S6). In concert with the pathway analysis results, T-cell related processes were the most enriched GO terms among DEIRGs being up-regulated in LUAD but repressed in LUSC (Figure 5C). On the other hand, genes being repressed in LUAD but up-regulated in LUSC were enriched of cell adhesion and cell proliferation related functions, which again supported the faster proliferation rate of LUSC (Figure 5E).

**Figure 6:**
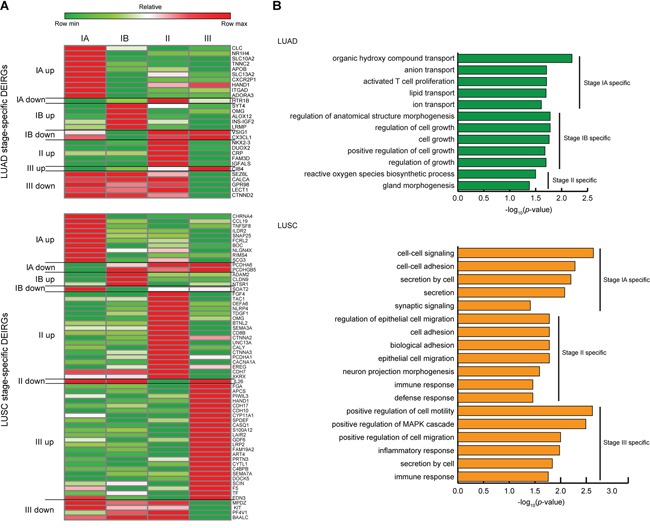
Expression profiles and GO analysis of the stage-specific DEIRGs in LUAD and LUSC **A.** Relative expression profiles of the stage-specific DEIRGs. Rows represent stage-specific DEIRGs with their gene symbols to the right, columns represent cancer stages. Up and down indicate the specifically up- and down-regulated DEIRGs at each stage, respectively. Heatmap is generated using the scaled relative abundance of log_2_-transformed RPKM ratios (tumor/normal). **B.** Enriched GO terms (*p*-value < 0.05, Fisher's exact test) of the stage-specific DEIRGs. X-axis represents log_10_-transformed *p*-values of GO term enrichment and y-axis stands for the enriched GO terms of the biological process category.

We next screened for DEIRGs with specific expression at certain cancer stage (Figure 6A, Supplementary Figure S6 and Supplementary Table S7). Using > 5 fold up- or down-regulation (FDR < 0.1) at one cancer stage and without > 2 fold up- or down-regulation (FDR < 0.1) at the other three stages, DEIRGs with either significant up-regulation or repression at any of the examined cancer stages were identified (Figure 6A). Enriched GO terms of these stage-specific genes also differed between LUAD and LUSC, and majority of the GO terms were related to the proliferation and metastasis features of cancer cells (Figure 6B).

## DISCUSSION

Increasing lines of evidence have shown that immune system plays an essential role in controlling cancer progression [19, 38, 39]. Although much efforts have been devoted to identify the causal factors and genes of lung cancers, how are immune-response related genes (IRGs) being regulated in different subtypes of lung cancers is still largely unknown. Here we systematically examined the expression changes of IRGs during the progression process of LUAD and LUSC, and identified that the expression profiles of IRGs could be a better classifier to distinguish LUAD and LUSC from normal tissues.

As the most common subtypes of NSCLC, LUAD and LUSC differ from each other in many aspects [9, 10]. Pathological studies have shown that LUSC grows faster than LUAD [8], but the underlining molecular mechanisms remain unclear. In concert with the clinical features, here we identified that cell cycle and cell proliferation related genes were up-regulated at earlier stage in LUSC than in LUAD, accompanied with the more severe repression of IRGs in LUSC than in LUAD. To be specific, KIFs and proteasome complex unit genes with cell cycle promoting functions had faster up-regulated expression in LUSC. On the other hand, MHC molecules and chemokines, which involved in immune response activation, were more rapidly repressed in LUSC. These results could explain the faster progression rate of LUSC as compared with LUAD.

We have also identified Toll-like receptor (TLR) and T cell receptor signaling pathways to be repressed more severely in LUSC than in LUAD. Reports have shown that the Toll-like receptors play a fundamental role in pathogen recognition and activation of innate immunity [40–42], and defects of the CD3-TCR complex could also facilitate tumor progression through immune evasion [43]. Thus, we speculated that more rapid repression of T cell mediated immune response may be correlated with the faster progression rate of LUSC.

In summary, our study revealed the differential expression profiles of IRGs during LUAD and LUSC progression, and identified significantly enriched expression change patterns as well as several other expression features of IRGs during cancer progression process. We discovered that the faster progression rate of LUSC may correlate with the faster activation of cell cycle promoting genes as well as the more rapid repression of immune system response. These results demonstrated the importance of IRGs in regulating the onset and progression of LUAD and LUSC, and may shed lights for the discovery of treatment methods for LUAD and LUSC.

## MATERIALS AND METHODS

### Data resources

The clinical information and RNA-Seq data of LUAD and LUSC patients were downloaded from TCGA database (Data version: 2014_06_14) through TCGA-Assembler (Version 1.0.3) (http://health.bsd.uchicago.edu/yji/TCGA-Assembler.htm) [44], using the commands TraverseAllDirectories, DownloadClinicalData, DownloadRNASeqData and the following parameters: assayPlatform ″RNASeqV2″, dataType ″rsem.genes.results″ and ″rsem.genes.normalized_results″.

### Sample collection and classification

RNA-Seq data in the TCGA database satisfying the following criteria were collected and used in this study: 1) samples were collected from the LUAD or LUSC patients; 2) RNA-Seq data of both the tumor and matched normal samples of the same patient should be available; 3) tumor samples had definitive tumor node metastasis (TNM) classification information. Both the LUAD and LUSC samples of selected patients were classified into five stages (IA, IB, II, III, and IV) according to the TNM information assigned by TCGA.

### Collection of immune-response related genes

The list of immune-response related genes (IRGs) was collected from the immunology database and analysis portal (IMMPORT) website (https://immport.niaid.nih.gov) [45], which contains genes either directly or indirectly correlated with the immune system. The expressed IRGs (with normalized counts ≥ 3 in at least one patient) were selected for further analysis.

### Identification of the differentially expressed immune-response related genes (DEIRGs)

The read count and RPKM matrix tables of IRGs were extracted from classified TCGA RNA-Seq data. IRGs with differential expression (DEIRGs) between the cancer and normal tissues were identified using edgeR (Version 3.10.0) with read count and RPKM matrix tables through the scripts ″run_DE_analysis.pl″ and ″analyze_diff_expr.pl″ with default parameters from Trinity software (Version 2.1.1) [46, 47]. The resulting *p*-values were subjected to Benjamini–Hochberg multiple testing correction to derive FDRs. Only genes with > 2 fold expression (tumor/normal) difference and FDR < 0.1 were considered for further analysis.

### Principle component analysis

The expressed IRGs and total genes (TGs) (with normalized counts ≥ 3 in at least one patient) were selected to conduct principle component analysis (PCA) by the prcomp function in R (Version 3.1.0) with the parameter ″scale = T″.

### Selection of DEIRGs with unidirectional expression changes in LUAD or LUSC

DEIRGs without significant down-regulation at any cancer stage and with > 2 fold up-regulation (FDR < 0.1) at one or more cancer stages were selected as unidirectional up-regulated DEIRGs. Conversely, DEIRGs without significant up-regulation at any cancer stage and with > 2 fold down-regulation (FDR < 0.1) at one or more cancer stages were selected as unidirectional down-regulated DEIRGs. The Venn diagram and pie chart representing the comparative results of DEIRGs with unidirectional expression changes were plotted using BioVenn, an online tool for comparison and visualization of biological lists (http://www.cmbi.ru.nl/cdd/biovenn/) [48].

### Identification of significantly enriched expression patterns

Significantly enriched expression patterns of DEIRGs were identified by the STEM (Short Time-series Expression Miner) software (Version 1.0) [29] using log_2_-transformed RPKM ratio (tumor/normal) values as input. Patterns with *p*-value < 0.05 (non-parametric clustering algorithm of STEM with Bonferroni correction) were identified as enriched patterns. Expression data from stage IV of neither LUAD nor LUSC were included in the analysis due to the limited number of patients (Supplementary Table S2).

### Comparative gene ontology enrichment analysis

Gene Ontology (GO) enrichment analysis of the common and specific DEIRGs was performed using DAVID Bioinformatics Resources 6.7 (http://david.abcc.ncifcrf.gov/) [49–51] supplemented by the REVIGO visualization toolbox [52]. Plots for enriched GO terms of DEIRGs were generated using the ggplot package (Version 2.1.0) in R [53]. GO enrichment analysis of DEIRGs with diverged expression patterns between LUAD and LUSC was performed by the ClueGO package (Version 2.2.5) of cytoscape (Version 3.3.0) [54, 55].

### Pathway enrichment analysis

Pathway enrichment analysis of DEIRGs with unidirectional expression changes in LUAD and LUSC was performed using EnrichNet (http://www.enrichnet.org/) [56–58]. Enriched genes in selected pathways were shown using the Pathview package (Version 1.8.0) in R [59, 60].

### Screen for DEIRGs with diverged or stage-specific expression patterns

DEIRGs with diverged expression patterns were obtained through comparing the unidirectional up-regulated genes in LUAD with the unidirectional down-regulated genes in LUSC, and *vice versa*.

DEIRGs with > 5 fold up-regulation (FDR < 0.1) at one cancer stage and without significant up-regulation at other cancer stages were selected as up-regulated stage-specific DEIRGs. Conversely, DEIRGs with > 5 fold down-regulation (FDR < 0.1) at one cancer stage and without significant down-regulation at other cancer stages were selected as down-regulated stage-specific DEIRGs. The heatmaps were generated using pheatmap package in R or GENE-E software (Version 2.1.134) (http://www.broadinstitute.org/cancer/software/GENE-E/). Expression data from stage IV of neither LUAD nor LUSC were included in the analysis due to the limited number of patients.

## SUPPLEMENTARY FIGURES AND TABLES












