# Supplementary material for: Differentiated regulation of immune-response related genes between LUAD and LUSC subtypes of lung cancers
Source: Oncotarget. 2016 Nov 15;8(1):133–44. doi: 10.18632/oncotarget.13346 (PMC5352059; doi:10.18632/oncotarget.13346)
Supplement: Supplementary file 1 [file oncotarget-08-133-s001.pdf]

Differentiated regulation of immune-response related genes between LUAD and LUSC subtypes of lung cancers

SUPPLEMENTARY FIGURES AND TABLES

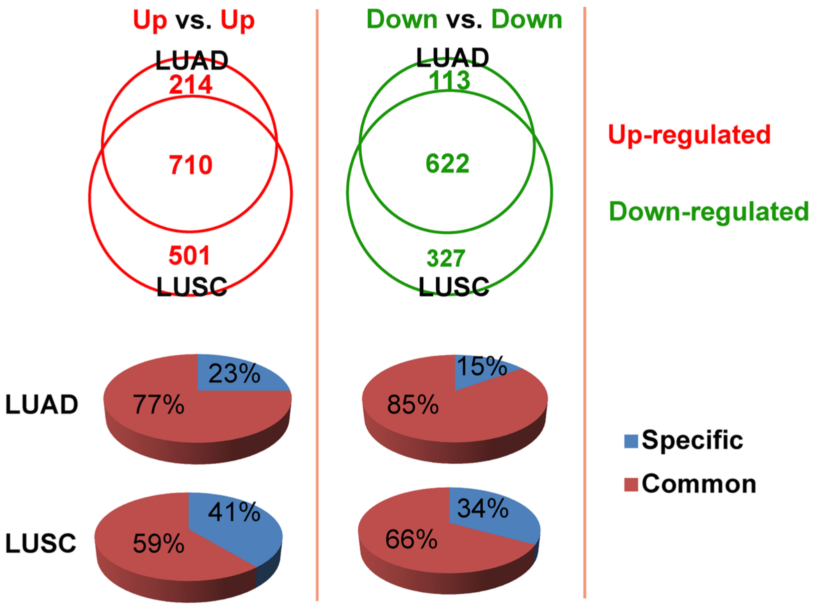

Supplementary Figure S1: Comparison of DEIRGs with unidirectional expression changes between LUAD and LUSC. The upper Venn diagrams present the No. of the common and specific DEIRGs being up-regulated (left) or down-regulated (right) in both LUAD and LUSC. The proportions of genes in each category are shown in the pie chart.

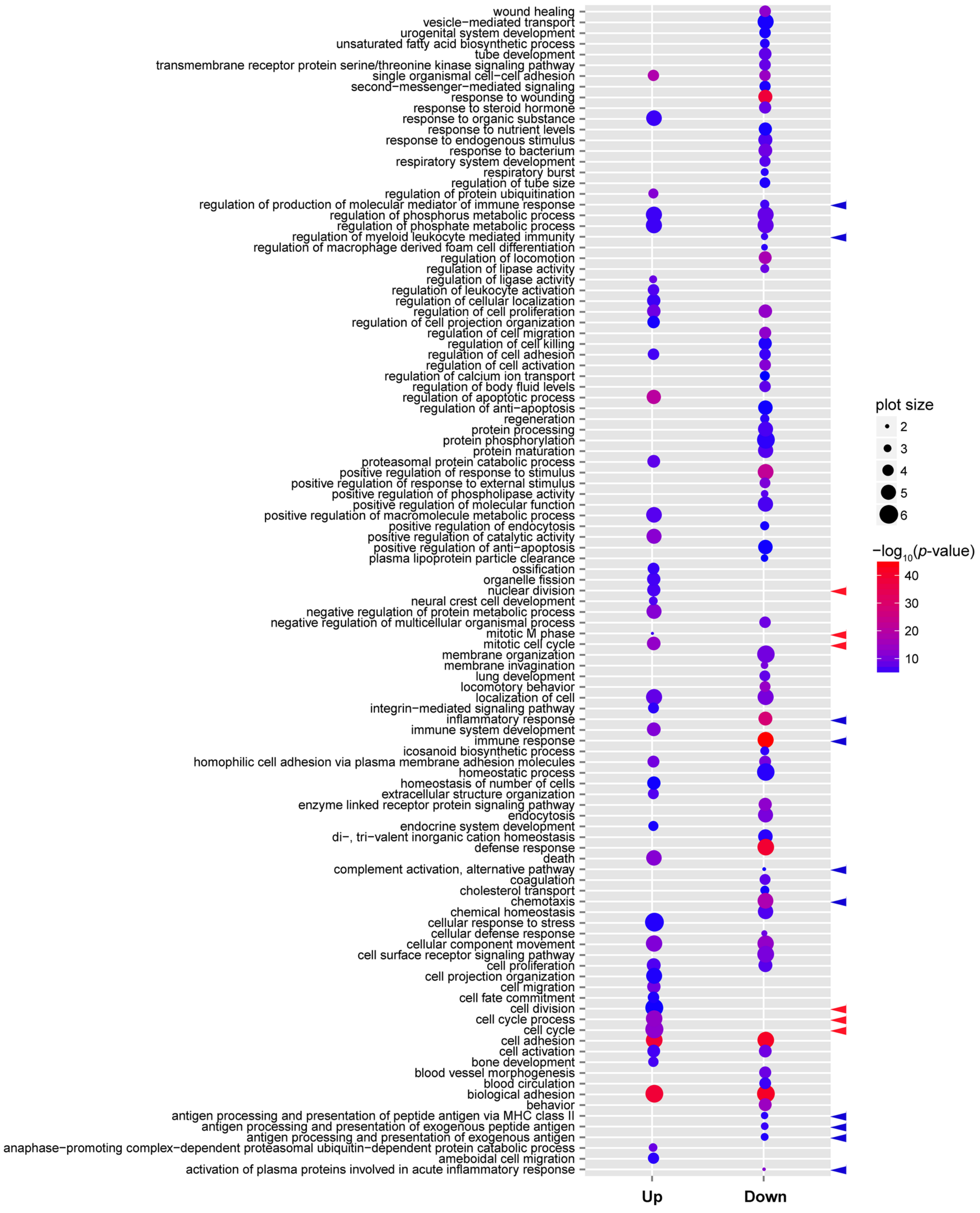

Supplementary Figure S2: GO analysis of the common up- (Up) or down-regulated (Down) DEIRGs with unidirectional expression changes in both LUAD and LUSC. Shown are the significantly enriched GO terms (FDR < 0.001, Fisher's exact test) of the biological process category. Dot size represents the frequency of the GO term in the GOA database. Dot color represents the $\log_{10}$-transformed enrichment *p*-value of each GO term. Up-regulated GO terms directly related to cell-cycle and down-regulated GO terms directly related to immune response are indicated by red and blue arrowheads, respectively.

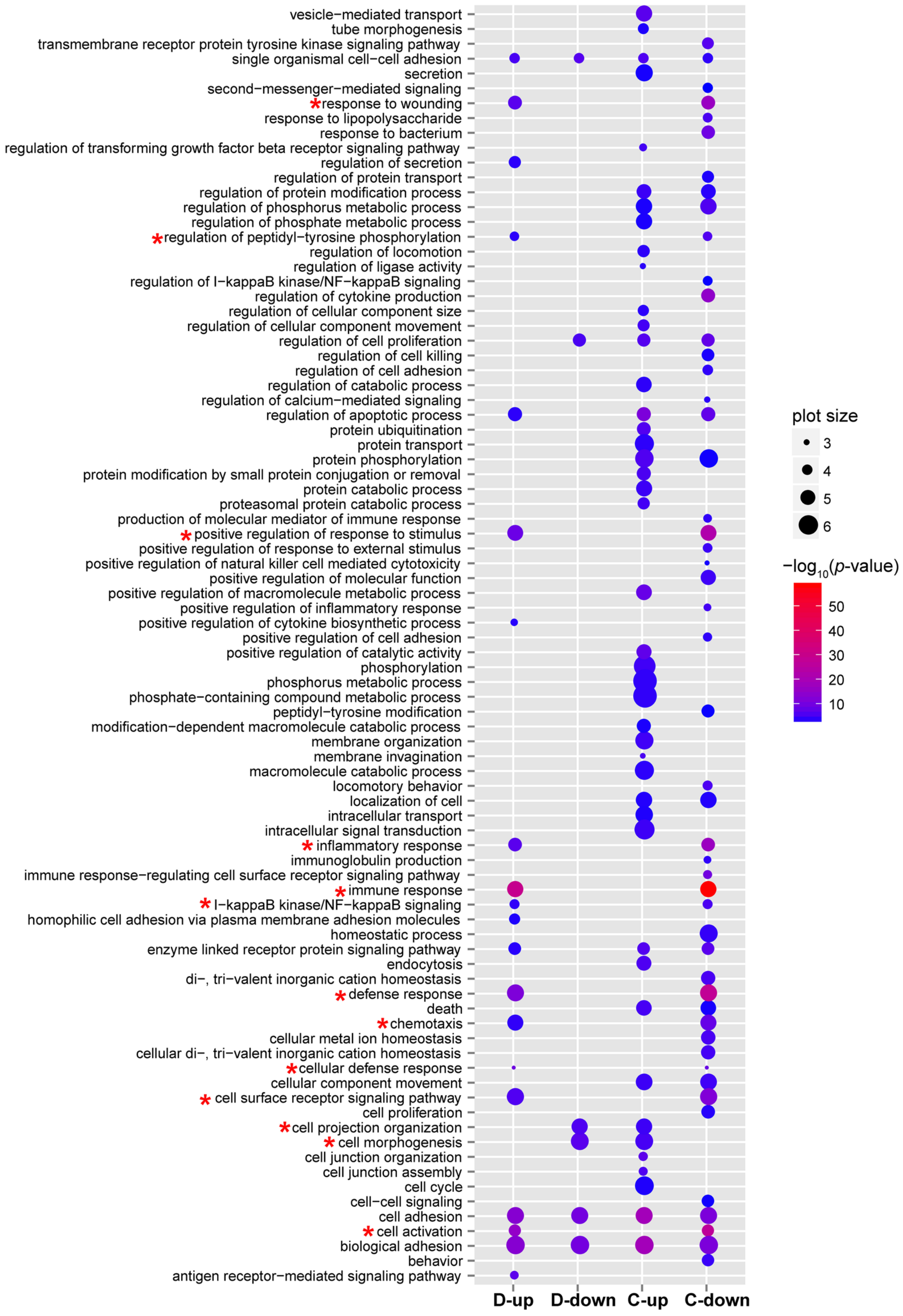

Supplementary Figure S3: GO analysis of the specific up- or down-regulated DEIRGs with unidirectional expression changes in LUAD (D-up or D-down) or LUSC (C-up or C-down). Shown are the significantly enriched GO terms (FDR < 0.001, Fisher's exact test) of the biological process category. Dot size represents the frequency of the GO term in the GOA database. Dot color represents the $\log_{10}$-transformed enrichment p-value of each GO term. GO terms among DEIRGs with diverged expression changes are marked by red asterisks.

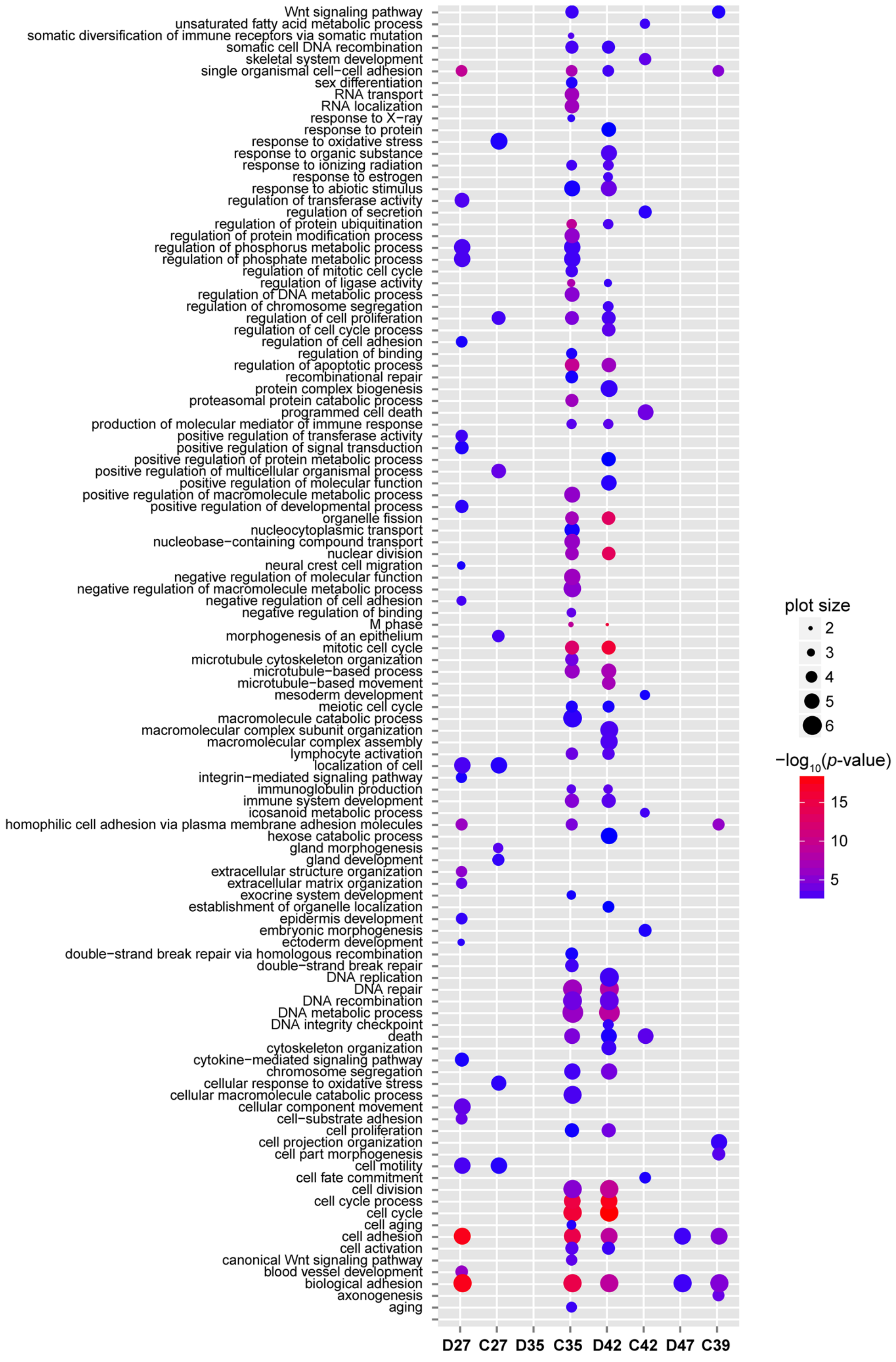

Supplementary Figure S4: GO analysis of DEIRGs with up-regulated expression patterns in LUAD (D27, D35, D42, and D47) or LUSC (C27, C35, C42, and C39). Shown are the significantly enriched GO terms (*p*-value < 0.01, Fisher's exact test) of the biological process category. Dot size represents the frequency of the GO term in the GOA database. Dot color represents the $\log_{10}$-transformed enrichment *p*-value of each GO term.

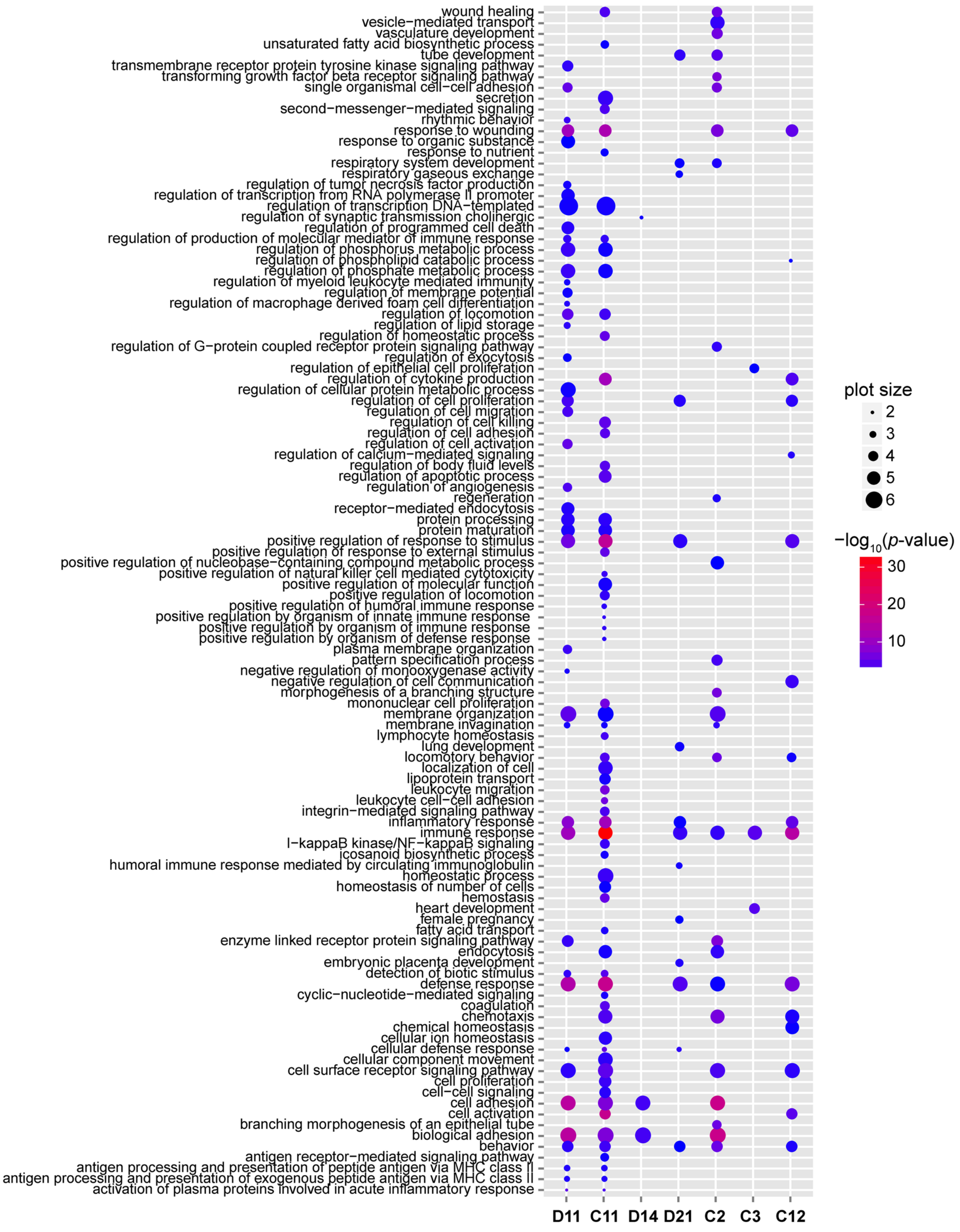

Supplementary Figure S5: GO analysis of DEIRGs with the down-regulated expression patterns in LUAD (D11, D14, and D21) or LUSC (C11, C2, C3, and C12). Shown are the significantly enriched GO terms (*p*-value < 0.01, Fisher's exact test) of the biological process category. Dot size represents the frequency of the GO term in the GOA database. Dot color represents the $\log_{10}$-transformed enrichment *p*-value of each GO term.

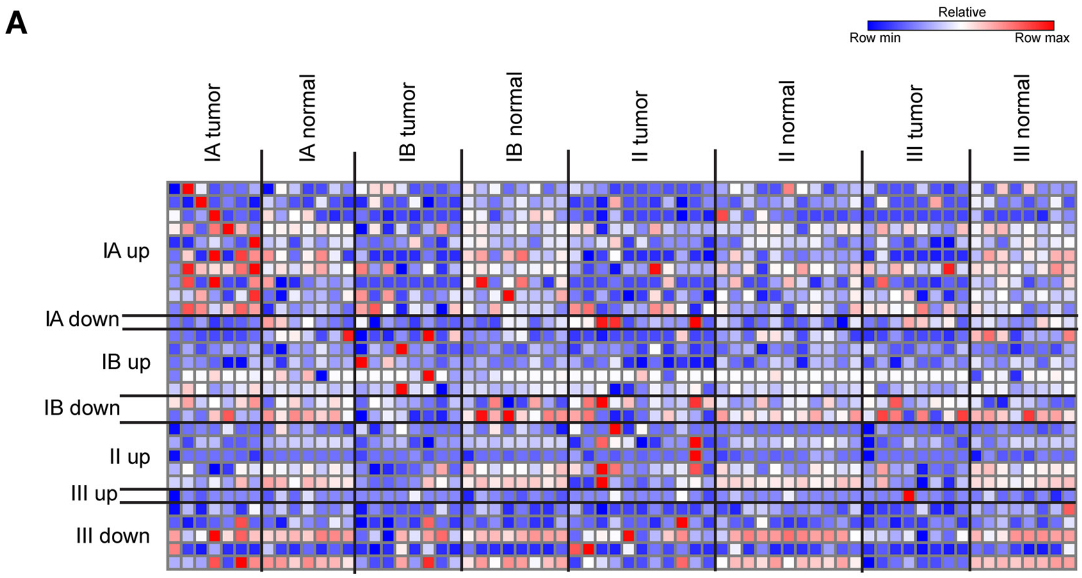

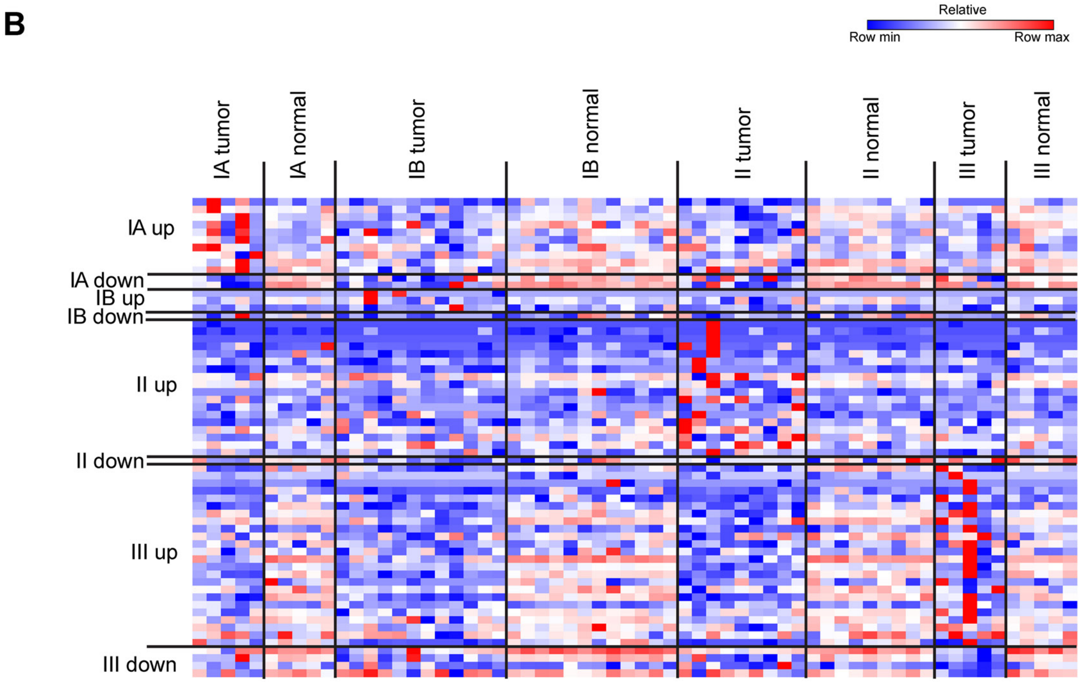

Supplementary Figure S6: Expression profiles of the up- or down-regulated stage-specific DEIRGs in LUAD (A) and LUSC (B) Rows represent up- or down-regulated stage-specific DEIRGs, and columns represent either tumor or normal samples at each cancer stage. Heatmap is generated using the scaled relative abundance of normalized count values of samples in each row.

Supplementary Table S1: Clinical information of patients with LUAD or LUSC (Modified from TCGA clinical data)

See Supplementary File 1

Supplementary Table S2: No. of patients with gene expression information of both the tumor and matched normal samples at each stage of LUAD and LUSC

Cancer stages	IA	IB	II	III	IV	Total
LUAD	7	8	11	8	2	36
LUSC	5	12	9	5	1	32

Supplementary Table S3: No. of DEIRGs with significantly enriched expression patterns in LUAD and LUSC

Significantly enriched expression patterns	No. of DEIRGs in LUAD	No. of DEIRGs in LUSC	No. of Common DEIRGs between LUAD and LUSC
**Pattern 27**	84	37	3
**Pattern 35**	2	319	0
**Pattern 42**	183	84	12
**Pattern 47**	42	0	0
**Pattern 39**	0	42	0
**Total number of genes among the up-regulated expression patterns**	311	482	136
**Pattern 11**	191	197	51
**Pattern 14**	23	0	0
**Pattern 21**	63	0	0
**Pattern 2**	0	150	0
**Pattern 12**	0	66	0
**Pattern 3**	0	32	0
**Total number of genes among the down-regulated expression patterns**	277	445	131

Supplementary Table S4: Expression information of the cell proliferation and cell cycle related genes with pattern 42 in LUAD and pattern 35 in LUSC. Log2 transformed RPKM ratio (tumor/normal) values of genes at stage (I-III) in LUAD and LUSC are listed

See Supplementary File 4

Supplementary Table S5: Expression information of the immune response related genes with patterns 11 and 21 in LUAD and patterns 11, 12, 2, and 3 in LUSC. Log2 transformed RPKM ratio (tumor/normal) values of genes at different stages (I-III) in LUAD and LUSC are listed

See Supplementary File 5

Supplementary Table S6: Expression information of DEIRGs with diverged expression changes between LUAD and LUSC. Log2 transformed RPKM ratio (tumor/normal) values of genes at different stages (I-III) in LUAD and LUSC are listed

See Supplementary File 6

Supplementary Table S7: Expression information of the stage-specific IRGs in LUAD and LUSC. Log2 transformed RPKM ratio (tumor/normal) values of genes at different stages (I-III) in LUAD and LUSC are listed

See Supplementary File 7
